# Implementation of Good Clinical Laboratory Practice (GCLP) guidelines within the External Quality Assurance Program Oversight Laboratory (EQAPOL)

**DOI:** 10.1016/j.jim.2013.09.012

**Published:** 2013-10-09

**Authors:** Christopher A. Todd, Ana M. Sanchez, Ambrosia Garcia, Thomas N. Denny, Marcella Sarzotti-Kelsoe

**Affiliations:** a Department of Surgery, Duke University Medical Center, Durham, NC, USA; b Duke Human Vaccine Institute, Duke University Medical Center, Durham, NC, USA; c Department of Immunology, Duke University Medical Center, Durham, NC, USA

**Keywords:** GCLP, Proficiency testing, Quality management, Quality assurance, Laboratory, Biorepository

## Abstract

The EQAPOL contract was awarded to Duke University to develop and manage global proficiency testing programs for flow cytometry-, ELISpot-, and Luminex bead-based assays (cytokine analytes), as well as create a genetically diverse panel of HIV-1 viral cultures to be made available to National Institutes of Health (NIH) researchers. As a part of this contract, EQAPOL was required to operate under Good Clinical Laboratory Practices (GCLP) that are traditionally used for laboratories conducting endpoint assays for human clinical trials. EQAPOL adapted these guidelines to the management of proficiency testing programs while simultaneously incorporating aspects of ISO/IEC 17043 which are specifically designed for external proficiency management. Over the first two years of the contract, the EQAPOL Oversight Laboratories received training, developed standard operating procedures and quality management practices, implemented strict quality control procedures for equipment, reagents, and documentation, and received audits from the EQAPOL Central Quality Assurance Unit. GCLP programs, such as EQAPOL, strengthen a laboratory's ability to perform critical assays and provide quality assessments of future potential vaccines.

## 1. Introduction

The External Quality Assurance Program Oversight Laboratory (EQAPOL) is a contract awarded by the National Institutes of Health/National Institute of Allergy and Infectious Diseases/Division of AIDS (NIH/NIAID/DAIDS) to support the development of external proficiency testing programs for flow cytometry-, ELISpot-, and Luminex bead-based assays (cytokine analytes). The EQAPOL Program is comprised of a Central Management Team, Central Quality Assurance Unit (CQAU), Statistical Group, Data Management Group, Biorepository, Central Laboratory, A3R5 Neutralizing Antibody Assay Validation Program, and three EQAPOL Oversight Laboratories (EOLs) described in detail in this issue of Journal of Immunological Methods (see Ferrari et al. for ELISpot; Staats et al. for ICS by Flow Cytometry; Sempowski et al. for cytokine-based Luminex). In addition to proficiency testing, EQAPOL is also tasked with creating a diverse panel of high-titer (approximately 10^9^ copies/mL), HIV-1 viral culture supernatants grown in PBMC from seed stocks (i.e., from plasma samples and other source material) using a Viral Diversity Core (see Sanchez et al. in this issue) and in validating immunogenicity assays (see Sarzotti-Kelsoe et al. in this issue).

The EQAPOL Laboratory Teams (EQAPOL Viral Diversity Core, Biorepository, Central Laboratory, A3R5 Neutralizing Antibody Assay Validation Program, and each of the EOLs) are required to operate under Good Clinical Laboratory Practices (GCLP), since this is a set of standards designed to facilitate uniform and consistent data generation and reporting. GCLP encompasses both quality assurance (QA) and quality control (QC) principles into its standards. QA proactively and periodically reviews the various components of the research process to assess adherence to standard operating procedures (SOPs) and policies and to determine the accuracy of research records. QC measures are continuous and carried out on all records (QC logs, worksheets, etc.) by the Laboratory Teams. While external laboratories, participating in the EQAPOL proficiency testing programs, are not required to operate under GCLP, many of these laboratories are already GCLP-compliant and perform clinical trial related work. It is for this reason that the program operates in GCLP compliance as it ensures the quality, integrity, and validity of the test data.

GCLP was initially designed by the British Association of Research Quality Assurance (BARQA) in 2003 and later expanded upon by the NIH/NIAID/DAIDS in 2008 to provide a regulatory framework to laboratories performing endpoint assays for HIV-1 human clinical trials (Stiles et al., 2003; Ezzelle et al., 2008). The two sets of GCLP guidelines were harmonized in 2009 in order to provide a single set of recommendations for laboratories to utilize (Sarzotti-Kelsoe et al., 2009). The process of converting laboratories into GCLP-compliant entities includes initial laboratory assessments and GCLP training; establishment of SOPs, Quality Management Systems and Study Plans; quality controlled equipment and reagents; optimization and validation of applicable assays; and laboratory audits and corrective action programs. The EQAPOL CQAU, which has over 10 years of experience in performing audits, document control and study monitoring in GCLP compliance, was tasked with implementing these standards for EQAPOL (Sarzotti-Kelsoe et al., 2009; Ozaki et al., 2012; Todd et al., 2012).

External Quality Assurance (EQA) Programs serve three purposes according to GCLP guidance: 1) provide a way for laboratories to ensure that data generated are timely, accurate, and clinically appropriate; 2) provide sponsors with assurance that data generated are of the highest quality; and 3) ensure that human specimens from clinical trials will be tested accurately and reliably (Ezzelle et al., 2008). Although GCLP is a robust set of guidelines governing the conduct of endpoint assays for clinical trials, there are no specific statements regarding the management of external proficiency testing programs. The International Organization for Standardization (ISO) and International Electrotechnical Commission (IEC) have created a set of guidelines/requirements (ISO/IEC 17043) for external proficiency testing programs to follow, so as to provide extra assurance to participants that the program is operated competently (ISO/IEC, 2010). ISO/IEC 17043 requirements primarily apply to management, planning and design, personnel, quality assurance, and confidentiality (ISO/IEC, 2010). The EQAPOL CQAU implemented GCLP, along with many aspects of ISO/IEC 17043, in an effort to make the program compliant to the most appropriate quality standards.

In addition to the proficiency testing programs, EQAPOL was also charged with establishing and characterizing clade-specific HIV-1 viral culture panels representing world-wide genetic diversity. These HIV-1 viral diversity panels are created from HIV-1 positive plasma specimens received from collaborators or from currently existing viral culture supernatants.

Finally, as an option exercised by the NIAID contract, EQAPOL was also charged with performing formal validation of specific immunogenicity assays to be employed as endpoint assays for HIV vaccine clinical trials. The Neutralizing Antibody Assay for HIV-1 in A3R5 cells was optimized and formally validated (Sarzotti-Kelsoe, et al. in this issue) under the oversight of the EQAPOL CQAU.

This report describes the process by which GCLP compliance was established for the entire EQAPOL Program.

## 2. Laboratory assessments

**Fig. 1:**
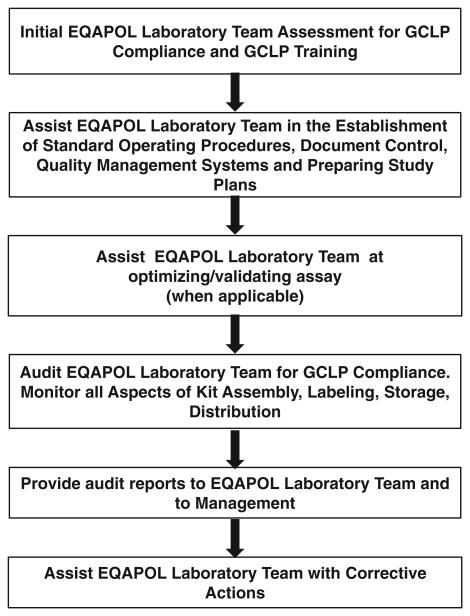
EQAPOL QAU approach to GCLP-compliance for EQAPOL Laboratory Teams. This schematic diagram illustrates the plan taken by the EQAPOL CQAU to assess the EQAPOL Laboratory Teams and bring them to GCLP-compliance.

Prior to implementing GCLP throughout the EQAPOL Program, the CQAU performed an overall assessment of each EQAPOL Laboratory Team. Fig. 1 illustrates the approach taken by the EQAPOL CQAU to bring each EQAPOL Laboratory Team into GCLP compliance. The laboratory assessment process did not apply to the A3R5 Validation Program as it was already operating under GCLP-compliance. The CQAU began the assessment process with an analysis of the organizational structure of the laboratory, provided documentation of critical areas that needed to be addressed in order to achieve GCLP compliance, created a general plan of action, and conducted GCLP training for all staff members of the laboratory. This introductory training to the GCLP guidelines discussed the requirements for organization and personnel training, equipment maintenance and calibration, reagent and specimen maintenance, document control, assay optimization and validation, and corrective action plans. Each EQAPOL Laboratory Team started the program with different levels of adherence to the principles of GCLP, thus laboratory-specific strategies were developed by the CQAU to address and close gaps in compliance based on previous experience from implementing GCLP in domestic and international laboratories (Ozaki et al., 2012; Gilmour et al., 2007). Successful establishment of GCLP compliance for the Laboratory Teams ranged from a minimum of three months for those with more quality experience to approximately two years.

## 3. Quality Management Systems

In order to effectively implement document and version control of SOPs, and manage training, equipment and calibration/maintenance records, the CQAU adopted a Quality Management System (QMS) (Q-Pulse, Gael Limited, Scotland) that allowed all of the aforementioned information to be captured and maintained electronically. The software package allows laboratory personnel to access SOPs on any computer with internal server access, which eliminates the need to have paper copies of SOPs present in the laboratories – further mitigating the risk of expired/uncontrolled procedures being used in the laboratory. The use of electronic copies also allows for better version control as paper copies may be altered while in use in the laboratory. In addition, the QMS allows the CQAU to electronically distribute SOPs to laboratory staff, maintain version control of documents, and manage SOP revision and training records.

The QMS has also been instrumental in streamlining the audit process by allowing auditors to complete a report and present findings electronically to each EQAPOL Program Team. The use of tablet computers running QMS-specific applications allows audits to be conducted in a more efficient manner in BSL-3 containment environments where removal of paper is restricted. Laboratory managers are able to view all audit findings and address them via corrective action entries. In order to ensure 21 CFR, Part 11 compliance (http://www.access.gpo.gov/nara/cfr/waisidx_05/21cfr11_05.html.), the QMS software utilizes an electronic signature package allowing users to enter their username and password to sign-off on training records, audit findings, and equipment maintenance records, which provides an audit trail.

## 4. Personnel organization and training

**Fig. 2:**
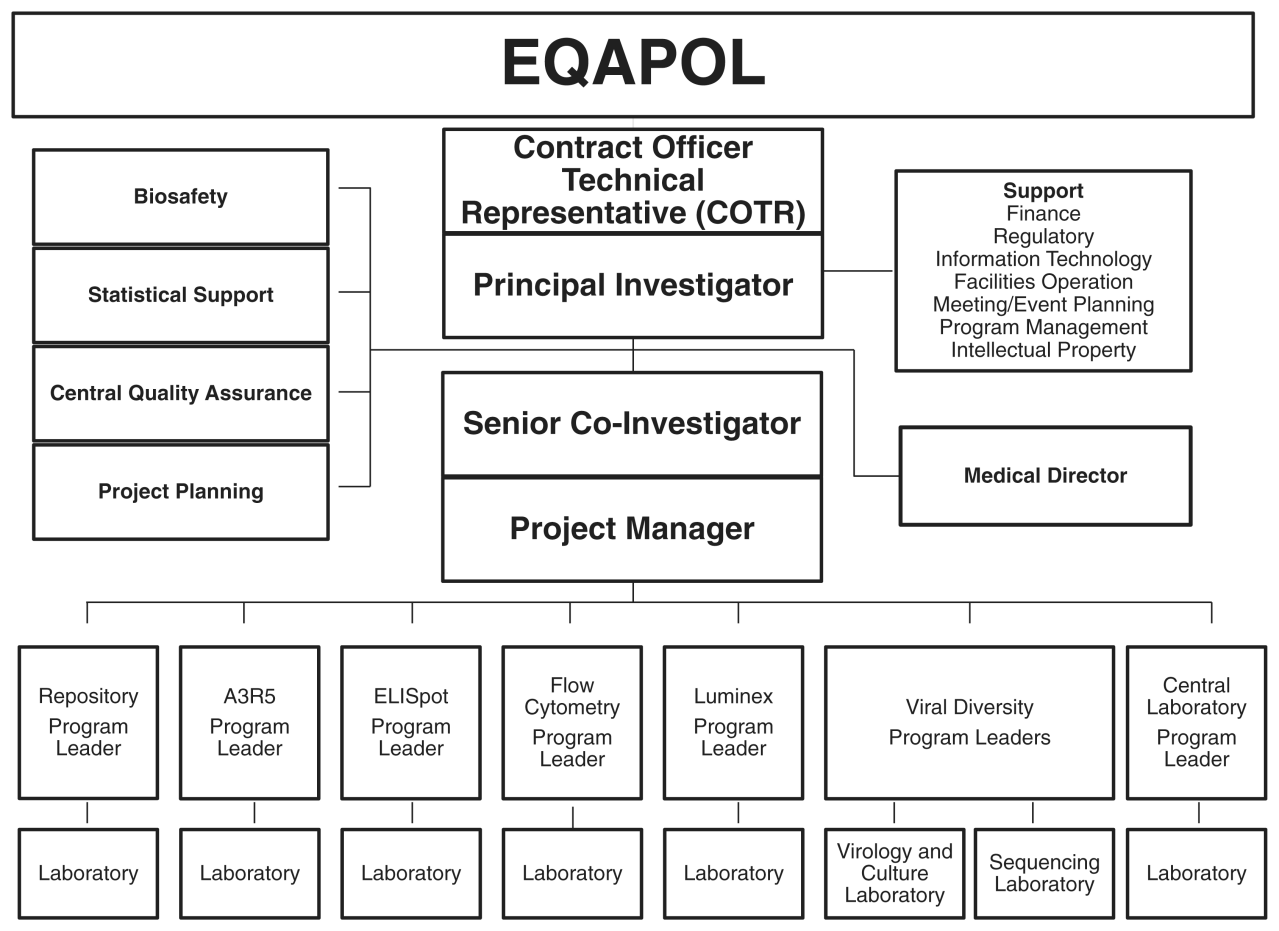
Example of organizational chart. This is an example of a GCLP-compliant organizational chart that represents the communication and reporting structure within the EQAPOL Program.

Formal reporting structures must be in place, in compliance with GCLP, to describe the relationships between the EQAPOL Management and the EQAPOL Laboratory Teams. Sollecito and Johnson describe communication as “necessary not only within the team but also between the team and the larger external environment, including other teams (Sollecito and Johnson, 2012).” In addition, errors or lack of communication and coordination have been identified as the key factors that lead to poor performance and detractors from quality (Sollecito and Johnson, 2012). EQAPOL Management created an organizational chart illustrating that relationship and each EQAPOL Laboratory Team also developed a chart describing the intra-laboratory management structure. Fig. 2 provides an example of an organizational chart that clearly delineates the reporting structure within EQAPOL.

According to GCLP, personnel must successfully complete required institutional, general safety and pathogen-specific training before performing laboratory assays. EQAPOL utilized the Duke University Occupational and Environmental Safety Office's on-line training program for laboratory, biological shipping, and other critical training (Chosewood et al., 2009; OSHA, 1992). Additionally, any laboratory personnel performing GCLP-compliant work must be able to document successful completion of assay training and provide evidence that he/she is competent by meeting pre-defined acceptance criteria. The EQAPOL CQAU helped laboratories design and implement SOPs for personnel training to assess initial and ongoing competency of the operators. Competency of operators must be assessed on a routine basis through annual intra-laboratory testing.

## 5. Equipment maintenance and calibration

Equipment must be verified that it is “fit for purpose” (Stiles et al., 2003) and must be maintained at high standards for use in GCLP-related activities. Documentation must be present to show that the equipment is properly installed, operated, inspected, cleaned, maintained, tested, and calibrated to ensure that all results are of optimal quality (Ezzelle et al., 2008; ISO/IEC, 2010). An essential component of the initial laboratory assessments was to identify the pieces of equipment that needed to meet these requirements based on risk assessment. For those items that needed to be formally validated, the CQAU assisted laboratory management in developing validation plans according to ICH guidelines (Guideline, 2010) and approving the validation before execution and upon completion. Validation of equipment helps assure that the item behaves accurately and consistently over time. Each piece of equipment used in EQAPOL receives a unique inventory number for quick reference in the equipment database. All EQAPOL Laboratory Teams manage their equipment in the QMS which captures the unique inventory number, model number, serial number, manufacturer, owner, equipment type, and location of the equipment. For the seven EQAPOL Laboratory Teams, approximately 250 pieces of equipment are currently catalogued and maintained under GCLP conditions. Additionally, equipment maintenance and calibration schedules (dependent upon the manufacturer's recommendations) can be entered for each equipment item. The QMS sends reminders to designated personnel for upcoming and/or overdue service for equipment. Data loggers are used by the EQAPOL Central Laboratory to monitor the environment of all temperature-sensitive shipments and provide assurance of the kit's quality to participating sites.

## 6. Standard operating procedure development

An elaborate SOP structure was developed for all of the EQAPOL Programs to help ensure that all procedures were conducted in an identical manner to guarantee the quality and integrity of generated data. GCLP guidelines require that SOPs be written in a standard format similar to the one recommended by the Clinical and Laboratory Standards Institute (CLSI) (Ezzelle et al., 2008). An SOP framework was already present in several EQAPOL Laboratory Teams, facilitating the process of implementation. SOPs are reviewed on a bi-annual basis with the EQAPOL CQAU managing version control. With an SOP system in place, the majority of SOPs that needed to be written were program-specific. For example, each program within EQAPOL developed SOPs on:

- how to conduct the assay,
- training for the assay,
- proper instrument use and maintenance,
- reagent bridging, and
- data analysis.

In addition to program-specific SOPs, EQAPOL Management developed SOPs that detailed how external proficiency (EP) kits are assembled and ordered using the EQAPOL web portal. While facilitating the control of SOPs, the QMS notified users of the effective dates for new SOPs and sent reminders when documents were due for review.

When conducting assays, EQAPOL laboratory operators are required to complete assay-specific checklists to document who performed each step. Assay checklists are associated to version controlled SOPs and accessible through the QMS. Checklists have been used in many industries as a way to improve quality and safety (Sollecito and Johnson, 2012). Recently, healthcare has also implemented checklists throughout many of its practices and an increasing amount of evidence has begun to show their importance (Sollecito and Johnson, 2012).

## 7. Study Plans

In order to conduct a GCLP compliant study and proficiency testing program, the Study Director needs to write a complete Study Plan outlining the scope and conduct of the study. Although EQAPOL is not fully adherent to ISO standards, the Study Plan incorporates many of ISO's concepts. EQAPOL Study Directors, Leadership, and the Statistical Group prepare program-specific Study Plans for each round of testing under the CQAU oversight. The EQAPOL Study Plans for proficiency testing program include the following:

- a detailed list of the program's organizational and management structure,
- introduction and background of the program,
- requirements for participation,
- number and type of program participants,
- proficiency panel contents,
- assay specifics,
- timeline for completion and upload of data,
- methods for data reporting,
- confidentiality, and
- methods for statistical analysis and grading.

EQAPOL Study Plans are approved and finalized by the EQAPOL Study Directors, Leadership, the CQAU Director, and the Statistician prior to the implementation of a particular EP send-out. Study Plans provide evidence that the EQAPOL EP was planned thoroughly and received Central Leadership approval prior to the conduct of the study. The Study Plans are not shared with the laboratories participating in the proficiency testing programs, as they contain information that could unblind their testing.

## 8. Audits

The EQAPOL CQAU performs internal and in-process audits of each EQAPOL Laboratory Team (at least annually) as required by GCLP guidelines to identify any potential gaps in compliance and/or deviations from established protocols/SOPs/Study Plans. Audits are performed by the EQAPOL CQAU with the assistance of other QA personnel not involved in the EQAPOL contract, to eliminate potential bias. Checklists are used throughout all audits and reports are prepared with any findings that need to be addressed. Laboratory personnel then respond to the audit findings with appropriate corrective actions to address the issues. The CQAU oversees the proficiency test kit assembly, reagent preparation, and shipment by the EQAPOL Central Laboratory, for at least one send-out per year for each program. As these are often the only proficiency testing materials that clinical trial immunogenicity endpoint assay laboratories receive, it is crucial that all aspects of the send-out are subject to CQAU oversight. At the conclusion of the audits, the CQAU prepares a report summarizing the kit preparation, send-out process and congruence with what is described in the Study Plan. Throughout the first series of audits, common findings existed for many of the EQAPOL Laboratory Teams. These non-conformances typically included a lack of a reagent bridging SOP, assay-specific training SOP, internal competency testing program, and SOPs for all pieces of equipment used during the conduct of the assay (Table 1).

## 9. Assay optimization, qualification, and validation

**Fig. 3:**
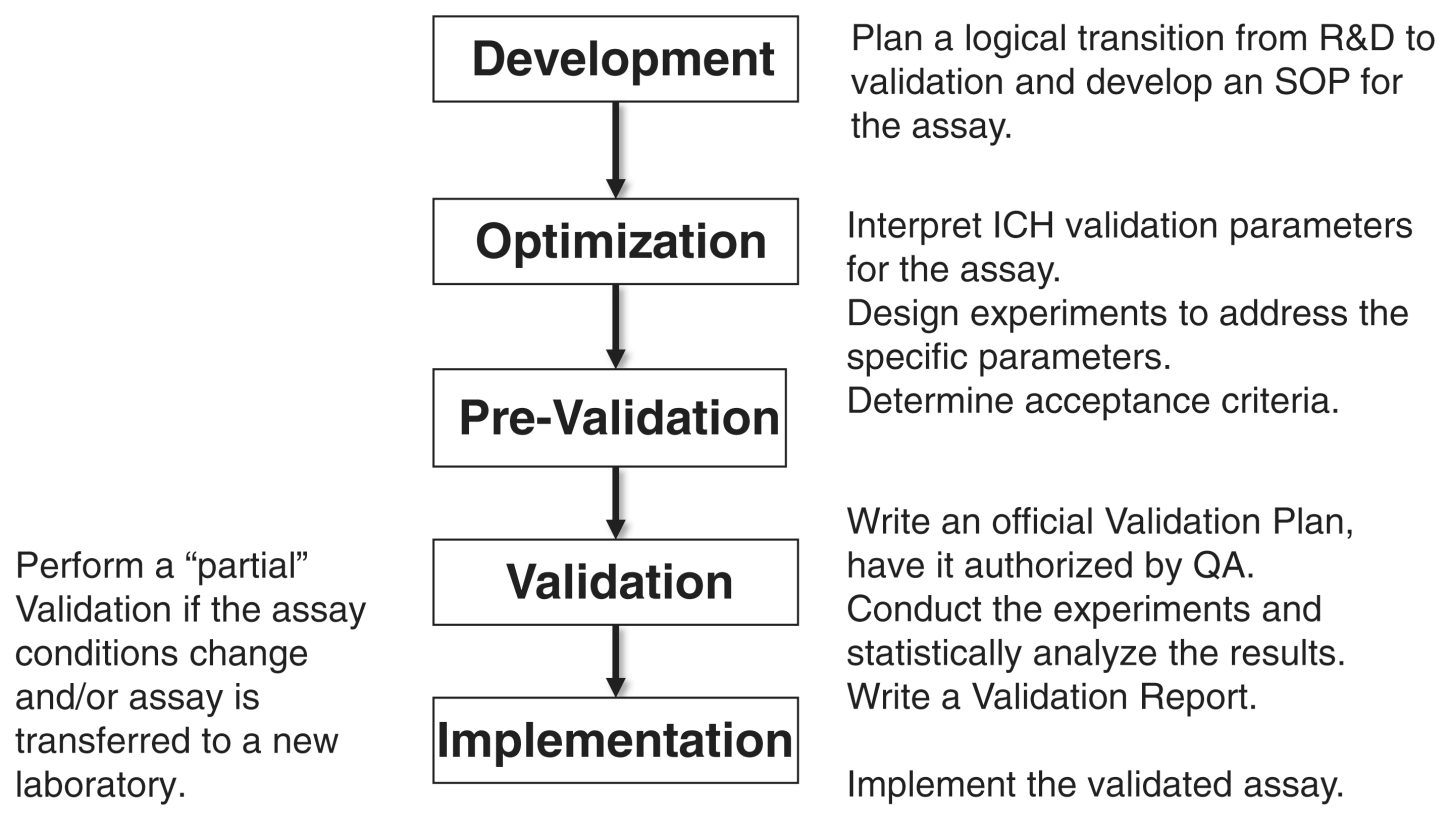
Assay development to validation. This schematic diagram illustrates the steps necessary to validate an endpoint immunogenicity assay such as the A3R5 Neutralizing Antibody Assay.

In previously reported studies, performed by the CQAU, it was demonstrated that it is easier to transfer an assay from one laboratory to another when the assay had been optimized and validated prior to the transfer (Ozaki et al., 2012). This process led to high reproducibility of results when inter-laboratory comparison was performed using a proficiency testing program (Todd et al., 2012). Validating an assay consists of analyzing the assay parameters recommended by the ICH-Q2 (R1) guidelines (Guideline, 2010): specificity, accuracy, precision, detection and quantitation limits, linearity, range, and robustness. As an option exercised by the NIAID contract, EQAPOL was charged with performing a formal validation of the Neutralizing Antibody Assay for HIV-1 in A3R5 cells. The process began with numerous optimization experiments to help establish pre-set acceptance criteria for the formal validation experiments (Fig. 3) (Sarzotti-Kelsoe et al., 2009). Applicable ICH parameters of validation were selected and a validation plan, inclusive of a statistical analysis plan, was written and authorized by the CQAU. The assay validation was performed, data statistically analyzed and a validation report was written and approved by EQAPOL Leadership, Study Director, Statistician and CQAU, and is reported in this issue (see Sarzotti-Kelsoe et al. in this issue). This validated assay is being transferred to and implemented by multiple laboratories and inter-laboratory reproducibility will be determined by developing a proficiency testing program for the A3R5 assay as a part of EQAPOL.

## 10. Reagent Biorepository Management

EQAPOL developed a Biorepository to manage all of the reagents and samples used in the External Quality Assurance (EQA) Programs, virus panel development, assay development, and validation. All materials are recorded electronically in the EQAPOL Application web-portal, which captures the reagent name, lot number, expiration date, and storage location.

The EQAPOL Biorepository uses an electronic, wireless temperature surveillance system for constant monitoring of all freezers, refrigerators, and incubators. Repository staff members receive alerts upon deviations from established temperature ranges for all monitored equipment. With this temperature monitoring software, the Biorepository manager and CQAU auditors are able to view and determine when out-of-range temperature excursions occurred, the operator who handled the excursion, and the corrective action taken to address the deviation. This information is stored permanently on the EQAPOL server which is backed up nightly and the back-ups are stored off-site. A Laboratory Data Management System (LDMS) is utilized for the inventory of peripheral blood mononuclear cells (PBMCs) that were processed from fresh leukapheresis blood units and all source material and viruses received/cultured as part of the EQAPOL Viral Diversity Program.

In accordance with GCLP guidelines, all new lots or batches of reagents and samples are parallel tested with previous lots to document their compatibility in the assay prior to being placed into use. Each EOL defined in SOPs the critical reagents required to be parallel tested as well as pass and fail criteria for their eligibility to be placed in proficiency testing kits. Examples of such reagents include PBMCs, Fetal Bovine Serum (FBS), and Streptavidin. Reagents that did not pass parallel testing were not permitted for use in the EQA proficiency test kits.

## 11. EQAPOL ordering, shipping and data reporting: web-based portal

As another aspect of the EQAPOL system, a user-friendly and comprehensive web-based portal was developed to secure all of the electronic data generated from the program. The web-based portal was developed specifically for the needs of EQAPOL by SciMed Solutions and operates in compliance with the Federal Information Security Management Act of 2002 (FISMA, 2006). Within the system, laboratories and individual users are given roles that are used to define which aspects of the system they can view. This is of particular importance for the EQA programs where sites should only see their own data.

The EQAPOL web portal is used to document all aspects of each EQA program from send-out of material information to data upload to EQA report retrieval. It maintains reagent and specimen inventory and location, as well as certificates of analysis for reagents. For each EQA send-out, the system records all samples/reagents sent to each site, shipment manifests, tracking information and shipment sent/receipt dates. For each EQA sendout, sites can download assay protocols and data reporting templates on the web-based system. Once a site completes an assay, the data reporting template is completed and returned using the web-based system. Sites also complete a post-assay questionnaire using the portal. As data are received from each site, the templates undergo data verification to ensure that the data are in the proper format prior to importing them into the EQAPOL database. Specific data verification instructions have been developed for each EQA program. The EQAPOL statisticians access all EQA data directly from this database ensuring data integrity throughout the system.

Once the statisticians complete all data analysis, site-specific reports are generated and uploaded to the web-based system with each site having access to their site report. Sites receive both a score out of 100 and a performance rating for each EP, with points awarded for different performance criteria (i.e., precision, accuracy, protocol adherence). These scores and performance ratings are entered into the web-based system to allow performance tracking over time. Finally, the web-based system can capture comments and files used as part of the remediation process. This comprehensive system allows for complete traceability of the EQA process.

The web portal also allows investigators to place orders for viral samples that are prepared by the EQAPOL Viral Diversity Core. Additionally, the portal captures virus culture and characterization data, and provides a means for approved laboratories to view virus inventory and order products. The system maintains an electronic inventory of all specimens and reagents for EQAPOL, and all applicable compliance information (Institutional Review Board, Material Transfer Agreement and Safety Compliance Forms) to ensure that sites only receive material which they are authorized to receive. These orders are then processed by EQAPOL Program Management and sent through an approval process.

## 12. Reporting to EQAPOL Leadership

A key aspect to maintaining GCLP-compliance within the EQAPOL Program is to keep Leadership abreast of key quality issues on a regular basis. The EQAPOL CQAU meets with the EQAPOL Steering Committee on a bi-weekly basis with updates on audits, SOP development, proficiency testing kit preparation and shipment status, and other key quality matters. The Steering Committee is comprised of the Program Director and Co-Director, Study Directors, IT Personnel, Finance, Regulatory Affairs Compliance, Statisticians, CQAU and other critical program managers. In addition, the CQAU presents annual progress updates to the EQAPOL External Scientific Advisory Board and receives feedback from the Board.

## 13. Archives

The EQAPOL CQAU provides archival functions for all study and proficiency testing materials. The materials are required to be kept in case a study needs to be investigated. Obsolete SOPs, study plans, raw data, and quality control documents are given to the EQAPOL CQAU and are stored in a space that has restricted access and is separated from the laboratory environment. Archived material is maintained indefinitely or until the Sponsor has requested destruction of the documents. In addition to on-site archiving, the CQAU has contracted with a third party sub-contractor to store documents off-site for extended periods of time.

## 14. Discussion

Implementation of GCLP into the EQAPOL Laboratory Teams began with an initial laboratory assessment, followed by GCLP training of laboratory staff, implementation of a QMS, equipment qualification, SOP document control, and audits by the CQAU. Converting EQAPOL into a GCLP-compliant operation has a number of added benefits that include increased credibility for the program and improved accuracy, integrity, and traceability of generated data. Having a CQAU in charge of monitoring the EQAPOL project has allowed for more stringent quality control measures of all processes within the program.

Implementing GCLP guidelines designed for clinical trial testing into a setting for proficiency testing was ultimately a challenge for the EQAPOL CQAU. Converting all of the EQAPOL Laboratory Teams into GCLP-compliant laboratories required approximately two years and multiple hurdles were encountered along the way. One of the primary challenges for the EQAPOL Program was unifying all of the programs under one system of SOPs and document control. Since EOLs were members of different departments within Duke, not all of them followed the same system of SOPs. To address this dilemma, the QAU utilized an electronic QMS which ultimately incorporated multiple sets of pre-existing SOPs. Additionally, the CQAU did eliminate SOP redundancies by creating an overarching system of SOPs that were to govern common processes across all programs.

SOP development for new GCLP assays was also a considerable hurdle because some of the laboratories lacked experience in the creation and usage of SOPs. Commitment from laboratories is typically weak at the beginning of GCLP conversion and leads to many delays in SOP finalization. The quicker a laboratory “bought-in” to the concept and importance of GCLP, the smoother the overall transition went. Throughout this two-year process, it became evident that those laboratories that had already implemented some of the key GCLP components into their operations were better prepared to become GCLP-complaint.

The up-front cost of operating a laboratory under GCLP is often seen as a hurdle, yet mistakes are often more costly then preventative measures (Crosby, 1979). Utilizing the EQAPOL contract, it has been possible to estimate that full implementation of GCLP into a laboratory increases operating costs by close to 20% (G. Sempowski, personal communication). This figure is based on financial estimates from laboratories with pre-existing quality control measures such as service contracts for equipment maintenance and calibration, standardized protocols, and reagent inventory systems. Pending funding approval, EQAPOL is considering ISO/IEC 17043 accreditation for the proficiency testing program. Although ISO/IEC accreditation is not a contractual requirement, it would add consistency and quality to all aspects of the program. Accreditation is a mechanism that would reassure external participants that quality and safety standards are demonstrated throughout the program (Sollecito and Johnson, 2012).

GCLP implementation and compliance in the EQAPOL laboratories have provided assurance that all processes are planned, performed, monitored, recorded, and reported in a reliable and consistent manner. Program participants can be assured that the kits are assembled with the utmost quality and detail and that all handling of data and statistical analysis had been thoroughly planned and executed. Ultimately, GCLP programs, such as EQAPOL, strengthen laboratory's ability to perform critical assays and provide quality assessments of potential vaccines in the future. Quality improvement within EQAPOL Laboratory Teams is an ongoing process and changes will continue to be made with future funding so that all aspects of the program can operate at the highest level of compliance possible.

## Figures and Tables

**Table 1:** Common audit findings and corrective actions.

Audit finding	Potential corrective actions
Lack of reagent bridging SOP	Develop SOP for parallel testing to ensure that all reagents used in kits perform equivalently across rounds
Lack of/inconsistent assay training	Develop SOP for training new employees and employees that have taken an extended absence Develop training matrix stating required SOPs
No internal competency testing	Develop program to assess each operator's ability to perform an assay
Lack of equipment SOPs	Develop common practices for using equipment, calibration schedules and preventative maintenance

## References

[R1] Chosewood LC, Wilson DE, Centers for Disease Control, Prevention (U.S.), National Institutes of Health (U.S.) (2009). Biosafety in Microbiological and Biomedical Laboratories.

[R2] Crosby PB (1979). Quality is Free: The Art of Making Quality Certain.

[R3] Ezzelle J, Rodriguez-Chavez I, Darden J, Stirewalt M, Kunwar N, Hitchcock R, Walter T, D'souza M (2008). Guidelines on good clinical laboratory practice: bridging operations between research and clinical research laboratories. J Pharm Biomed Anal.

[R4] (2006). Federal Information Security Management Act. 44 U.S.C. §§ 3541-3549.

[R5] Gilmour JW, Stevens WS, Gray C, de Souza M (2007). Laboratory expansion to large-scale international HIV preventive vaccine trials. Curr Opin HIV AIDS.

[R6] Guideline, I.H.T. (2010). Validation of Analytical Procedures: Text and Methodology Q2 (R1) (2005). http://www.ich.org/cache/compo/363-272-1.html.

[R7] ISO/IEC (2010). Conformity Assessment – General Requirement for Proficiency Testing. 17043.

[R8] OSHA (1992). Occupational exposure to bloodborne pathogens; approval of information collection requirements – OSHA. Final rule; Approval of Information Collection Requirements. Federal register.

[R9] Ozaki DA, Gao H, Todd CA, Greene KM, Montefiori DC, Sarzotti-Kelsoe M (2012). International technology transfer of a GCLP-compliant HIV-1 neutralizing antibody assay for human clinical trials. PLoS ONE.

[R10] Sarzotti-Kelsoe M, Cox J, Cleland N, Denny T, Hural J, Needham L, Ozaki D, Rodriguez-Chavez IR, Stevens G, Stiles T (2009). Evaluation and recommendations on good clinical laboratory practice guidelines for phase I–III clinical trials. PLoS Med.

[R11] Sollecito WA, Johnson JK (2012). Mclaughlin and Kaluzny's Continuous Quality Improvement in Health Care.

[R12] Stiles TG, Mawby V, Barqa N (2003). Good Clinical Laboratory Practice (GCLP): A Quality System for Laboratories that undertake the Analyses of Samples from Clinical Trials.

[R13] Todd CA, Greene KM, Yu X, Ozaki DA, Gao H, Huang Y, Wang M, Li G, Brown R, Wood B, D'Souza MP, Gilbert P, Montefiori DC, Sarzotti-Kelsoe M (2012). Development and implementation of an international proficiency testing program for a neutralizing antibody assay for HIV-1 in TZM-bl cells. J Immunol Methods.

